# Prevalence of *Salmonella* Typhimurium in Foods, Animals, and Human Origin in Iran: A Systematic Review and Meta‐Analysis

**DOI:** 10.1002/hsr2.71158

**Published:** 2025-09-09

**Authors:** Negar Narimisa, Shabnam Razavi, Faramarz Masjedian Jazi

**Affiliations:** ^1^ Microbial Biotechnology Research Center Iran University of Medical Sciences Tehran Iran; ^2^ Department of Microbiology, School of Medicine Iran University of Medical Sciences Tehran Iran

**Keywords:** databases, Iran, meta‐analysis, prevalence, *Salmonella* Typhimurium

## Abstract

**Background and Aims:**

*Salmonella enterica* is a globally significant foodborne pathogen, with Typhimurium being one of the most prevalent serotypes linked to foodborne illnesses. Despite its importance, there is a notable absence of meta‐analytical studies examining the prevalence of this bacterium in Iran. This meta‐analysis aims to assess the prevalence of *S*. Typhimurium in food, animals, and human populations in Iran.

**Methods:**

Studies on the prevalence of *S*. Typhimurium published from 1936 to December 2023 were gathered from databases including PubMed, Scopus, Web of Science, and SID. The data collected were analyzed using Stata software.

**Results:**

A total of 52 studies met the inclusion criteria for the analysis of *S*. Typhimurium prevalence in clinical and environmental isolates in Iran. The overall prevalence of *S*. Typhimurium in total isolates from food, animals, and human sources was determined to be 4% (95% CI: 3%–6%). Additionally, the analysis revealed a nearly stable trend in the prevalence of *S*. Typhimurium over the years.

**Conclusion:**

The relatively high prevalence of *S*. Typhimurium in animal isolates underscores the necessity for implementing stricter infection control measures. Furthermore, it is essential to establish appropriate diagnostic criteria and management guidelines for screening this pathogen across various sample types to mitigate its spread.

## 1 Introduction

*Salmonella* is a Gram‐negative facultative anaerobic bacterium that belongs to the Enterobacteriaceae family. While *Salmonella* species are widespread in the environment, their main habitat is the intestinal tract of animals [1, 2]. *Salmonella* is an important foodborne pathogen that is estimated to cause 115 million human infections and 370,000 deaths globally each year [3, 4]. Infections can occur through the consumption of contaminated food (such as eggs, milk, and poultry) or water, contact with infected animals, or international food trade. The burden of infectious diseases extends beyond morbidity and mortality in humans and animals, also affecting trade and causing socioeconomic problems [5, 6]. Infected animals may exhibit a range of symptoms, from mild gastroenteritis to severe cases that could ultimately result in death [7].

The genus *Salmonella* consists of two species: *S*. *enterica* (with six subspecies) and *S*. *bongori* (non‐subspecies). *Salmonella enterica* is composed of six subspecies: *S. enterica* subsp. *enterica*, *S. enterica* subsp. *salamae*, *S. enterica* subsp. *arizonae*, *S. enterica* subsp. *diarizonae*, *S. enterica* subsp. *houtenae*, and *S*. *enterica* subsp. *indica* [8]. The most clinically significant subspecies is *S*. *enterica* subspecies Enterica, which can cause infection in humans and warm‐blooded animals. It includes approximately 1500 serovars, all of which have the potential to be pathogenic to humans. Among these serovars, approximately 80 serovars are responsible for around 99% of *Salmonella* infections in humans and animals [9, 10].

Non‐typhoidal *Salmonella* such as *Salmonella enterica* serotype Typhimurium is a significant enteric infection in humans, especially among neonates and young children [11]. The morbidity and mortality associated with *Salmonella* infections pose a considerable burden in both developing and developed nations [12]. The rise of antibiotic‐resistant foodborne pathogens has heightened public concern, as these strains exhibit increased virulence, leading to higher mortality rates among affected individuals [13].

*Salmonella enterica* serovar Enteritidis and serovar Typhimurium are the predominant agents responsible for non‐typhoidal salmonellosis in humans. Serovar Typhimurium is now recognized as one of the most prevalent *Salmonella* serotypes linked to human infections [14, 15]. In Europe, it ranks as the most frequently identified serovar among isolates from humans, pigs, and pork. Conversely, in the United States, it is among the top five serovars associated with human cases of salmonellosis [16, 17, 18].

Despite improvements in food hygiene and safety standards, infections caused by this bacterium remain a common public health problem worldwide [19]. Therefore, this study aims to analyze the prevalence of *S*. Typhimurium isolated from animals, food, and humans in Iran using a meta‐analysis approach. The results of this study can help to understand the prevalence and origin of *S*. Typhimurium in Iran, which in turn can be useful for eradicating and preventing diseases caused by *S*. Typhimurium.

## 2 Material and Method

### 2.1 Search Strategies

A systematic literature search was performed across Web of Science, PubMed, Scopus, and SID, covering the period from 1936 to December 2023. The search strategy employed the terms (“*Salmonella* Typhimurium” OR “*S*. Typhimurium”) AND (Iran). This review was carried out and reported in accordance with the Preferred Reporting Items for Systematic Reviews and Meta‐Analyzes (PRISMA) guidelines [20]. All identified articles were compiled using EndNote X20 Citation Manager Software, and duplicates were eliminated before the review process. The remaining citations were subsequently uploaded to Rayyan, a citation classification application [21].

### 2.2 Eligibility Criteria

Two reviewers independently evaluated titles, abstracts, and full texts to identify studies that met the inclusion criteria. Any discrepancies were resolved through consensus.

The studies included in this review were selected based on the following criteria: (1) Original articles reporting the prevalence of *S*. Typhimurium isolates in food, animal, and human samples, either relative to the total isolates or the total *Salmonella* serotypes identified; (2) studies conducted in Iran; and (3) studies published in either Persian or English.

Studies were excluded based on the following criteria: (1) studies that did not provide the exact number of *S*. Typhimurium isolates; (2) duplicate reports; (3) review; (4) case reports; (5) clinical trial studies; (6) short communications; (7) conference papers; (8) letters; (9) book chapters; and (10) studies published in languages other than English or Persian.

### 2.3 Quality Assessment

The quality of eligible studies was assessed independently by two authors using the prevalence Joanna Briggs Institute (JBI) Critical Appraisal Checklist [15]. This checklist comprises nine questions that evaluate research quality, focusing on appropriate sampling techniques, research objectives, and data analysis methods. Each item is rated as “yes,” “no,” or “unclear.” A “yes” response receives a score of 1 point, while “no” or “unclear” responses receive 0 points. The average score for each paper was assessed independently by two reviewers, with any disagreements resolved through consensus or discussion with a third author. Studies scoring 7 or higher were classified as high quality, those scoring between 5 and 7 as medium quality, and studies scoring 4 or lower as low quality.

### 2.4 Data Extraction

For each included study, the following details were extracted: the first author's name, publication year, sample size, source of isolation, method of *S*. Typhimurium detection, and the province and regions from which the isolates were collected (Table 1). To estimate the overall prevalence through meta‐analysis, we utilized the “metaprop” package in STATA version 17.0 (STATA, College Station, TX, USA) [7]. The pooled prevalence of *S*. Typhimurium across various samples, alongside a 95% confidence interval, was calculated using the Freeman‐Tukey double arcsine transformation within a random‐effects model.

**Table 1:** Characteristics of included studies.

First author	Quality	Publication year	Province	Region	Source	Origin	Method	Total	*Salmonella*	*Salmonella* Typhimurium
Moosavy [22]	8	2015	East Azarbayjan	Northwest	Food	Egg	Multiplex PCR	150	2	1
Nosrati [23]	8	2012	Tehran	Center	Meat	Food (cow meat)	PCR	170	3	2
Basti [24]	7	2004			Animal	Fish	Serologically	120	5	1
Jamshidi [25]	8	2008	Khorasan	Northeast	Animal	Poultry carcasses	Multiplex PCR	60	5	1
Ranjbari [26]	8	2015	Fars	Center	Food	Duck eggs and turkey	PCR	300	7	7
Tajbakhsh [27]	7	2015	Chaharmahal va Bakhtiari	Southwest	Food	Food (Processed food)	PCR	50	9	1
Askari [28]	9	2020	Tehran	Center	Animal	Dog	PCR	200	11	7
Karimi [29]	8	2013	Kurdistan	Northwest	Meat	Meat (cow)	Multiplex PCR	60	12	4
Asma Afshari [30]	6	2017	Khorasan	Northeast	Animal	Broiler	Multiplex PCR	100	14	5
Monadim [31]	6	2014	Kohgiluyeh and Boyer‐Ahmad	Southwest	Food	Egg	Multiplex PCR	210	14	12
Zare [32]	5	2015	East Azarbayjan	Northwest	Animal	Domestic	Serologically	250	15	5
STAJI [33]	8	2017	Semnan	Center	Animal	Duck	Multiplex PCR	247	18	10
Rahimi [34]	7	2011			Animal	Seafood	Serologically	384	19	4
Tajbakhsh [35]	8	2013	Chaharmahal va Bakhtiari	Southwest	Animal	Cow, Sheep and Goat's Milk	PCR	1100	20	7
Khakrizi [36]	8	2022	Tehran	Center	Animal	Pet dogs	Serologically	256	21	4
Nazari [37]	7	2017			Meat	Camel meat	PCR	150	22	3
Bokaeian [38]	5	2006	Sistan and Baluchestan	Southeast	Animal	Chicken (skin, meat,.)	Serologically	250	28	4
Momtaz [39]	7	2014	Chaharmahal va Bakhtiari	Southwest	Meat	poultry meat	PCR	620	28	12
Manafi [40]	9	2020	West Azerbaijan	Northwest	Meat	Meat	Multiplex PCR	120	29	3
Moghadam [41]	7	2022	Alborz	Center	Animal	livestock	Serologically		30	4
Salehi [42]	8	2006			Animal	Bovine	Serologically	400	33	22
Moghadam [43]	9	2023	Chaharmahal va Bakhtiari	Southwest	Meat	Poultry meat	Multiplex PCR	400	36	36
Namroodi [44]	5	2016			Animal	Dog	Serologically	210	40	14
Shimi [45]	6	1997			Animal	Cat	Serologically	301	42	16
Firouzabadi [46]	8	2019	Kerman	Southeast	Animal	Broiler chickens	PCR	110	53	26
Akbarmehr [47]	7	2010	East Azarbayjan	Northwest	Animal	Poultry	Multiplex PCR	634	58	13
Firoozeh [48]	8	2011	Tehran	Center	Human	Human	Multiplex PCR		58	5
Fardsanei [49]	8	2021			Human	Human	Multiplex PCR	2116	59	11
Siasi [50]	8	2020	Tehran	Center	Human	Human	Serologically	676	60	30
Ranjbar [51]	7	2017	Tehran	Center	Human	Human	Serologically		68	21
Ezatpanah [52]	7	2013	Markazi	Center	Animal	Chicken	Serologically	245	75	4
Mehrabian [53]	7	2007	Tehran	Center	Meat	Meat	Serologically	400	80	12
Bonyadian [54]	6	2006	Yazd	Center	Animal	Chicken carcasses	Serologically	435	90	47
Abdollahi [55]	7	2011			Human	Human	Serologically		96	35
Alzwghaibi [56]	8	2018	Tehran	Center			Multiplex PCR		100	32
Moghadam [57]	7	2017	Kerman	Southeast	Human	Human	PCR	1125	130	29
Ranjbar [58]	6	2011	Tehran	Center	Human	Human	Serologically	5900	139	20
Doosti [59]	9	2016	Chaharmahal va Bakhtiari	Southwest	Animal	Poultry	Multiplex PCR	300	245	138
Doosti [60]	9	2016			Animal	Poultry	PCR	600	287	49
Mashouf [61]	5	2007	Hamadan	Center	Human	Human			296	57
MOGHADDAM [62]	6	1990	Tehran	Center	Human	Human	Serologically		508	232
Khaltabadi [63]	7	2019					PCR		884	150
Azizpour [64]	8	2021	Ardebil	Northwest	Meat	Chicken Meat	PCR	100		1
Dilmaghani [65]	7	2011			Animal	Avians	Multiplex PCR	1,870		52
Halimi [66]	8	2014	Khorasan	Northeast	Animal	Cow	PCR	332		13
JAMSHIDI [67]	7	2010	Khorasan	Northeast	Food	Egg	Multiplex PCR	250		4
Keshmiri [68]	9	2022					Serologically	31		11
NAZER [69]	6	1994	Fars	Center	Food	Egg	Serologically	100		10
Rahimi [70]	8	2021	Qazvin	Center	Food	Egg	Real‐Time PCR	20		4
Ranjbar [71]	6	2013	Tehran	Center	Human	Human	Serologically	650		21
Estabergi [72]	7	2020			Food	Food	Biochemical	138		12
Moghadam [73]	6	2017	Kerman	Southeast	Human	Human	PCR	891		48

The heterogeneity among the studies included in the meta‐analysis was assessed using the I² statistic. An I² value of ≤ 25% indicates low heterogeneity, values between 25% and 75% indicate moderate heterogeneity, and values exceeding 75% suggest high heterogeneity.

Subgroup analyzes were conducted based on publication year, city of study, source of isolation, type of animal from which the isolate was obtained, and the method used for detecting *S*. Typhimurium.

To assess publication bias, we utilized funnel plots. This method generates a scatterplot with effect sizes on the horizontal axis and a measure of each study's size on the vertical axis [74]. We also applied Begg's rank correlation test, which evaluates potential publication bias by examining the correlation between the ranks of effect estimates and their variances [75]. Additionally, we employed Egger's regression test, which performs a linear regression of the intervention effect estimates based on their standard errors, weighted by inverse variance. A *p*‐value of less than 0.05 was deemed indicative of publication bias [76, 77].

Furthermore, we conducted a sensitivity analysis to determine the robustness of the model, checking whether specific studies influenced the results or if other potential sources of bias were present [78].

## 3 Results

### 3.1 Search Results

A total of 1105 studies were initially retrieved. After removing duplicates using EndNote software, 655 studies were screened, resulting in a full‐text examination of 83 studies that reported the prevalence of *S*. Typhimurium. Ultimately, 52 studies met the eligibility criteria and were included in the meta‐analysis.

**Figure 1:**
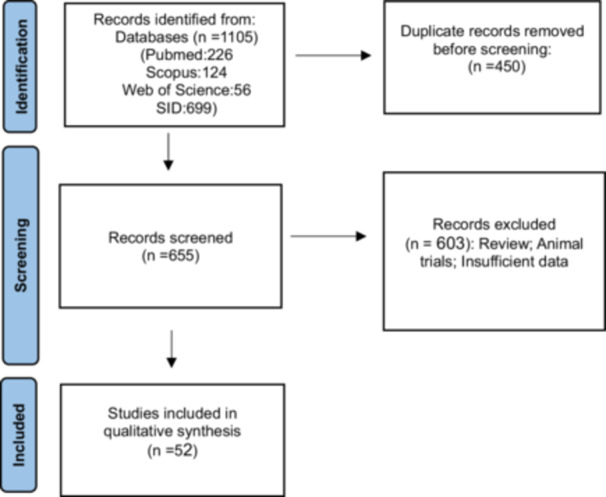
The study prisma flow diagram.

Figure 1 illustrates the studies selection process in accordance with the Preferred Reporting Items for Systematic Reviews and Meta‐Analyzes (PRISMA) guidelines. Table 1 presents the characteristics of the included studies along with their quality assessment scores. Of the total studies reviewed, 39 were classified as high quality, while 13 were deemed medium quality (Table 1).

### 3.2 Meta‐Analysis

#### 3.2.1 Prevalence of *S*. Typhimurium in Total Isolates

**Figure 2:**
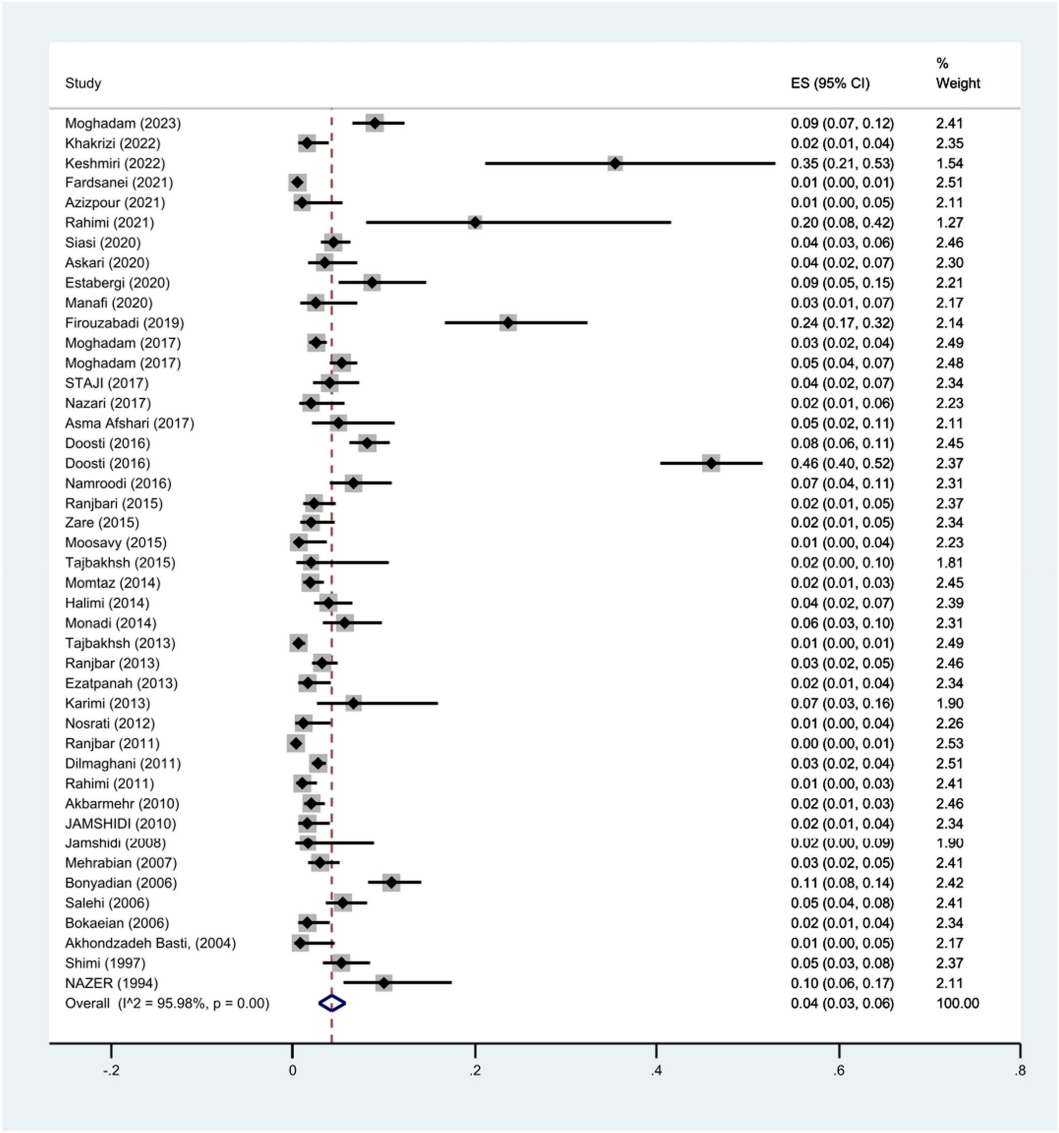
Forest plot showing the prevalence of *S*. Typhimurium in total isolates.

This study included 44 studies from 52 included studies reported the prevalence of *S*. Typhimurium in total isolates. The prevalence of *S*. Typhimurium in these isolates was 4% (95% CI: 3%–6%; I^2^ = 95.98%; *p *< 0.001) (Figure 2).

**Figure 3:**
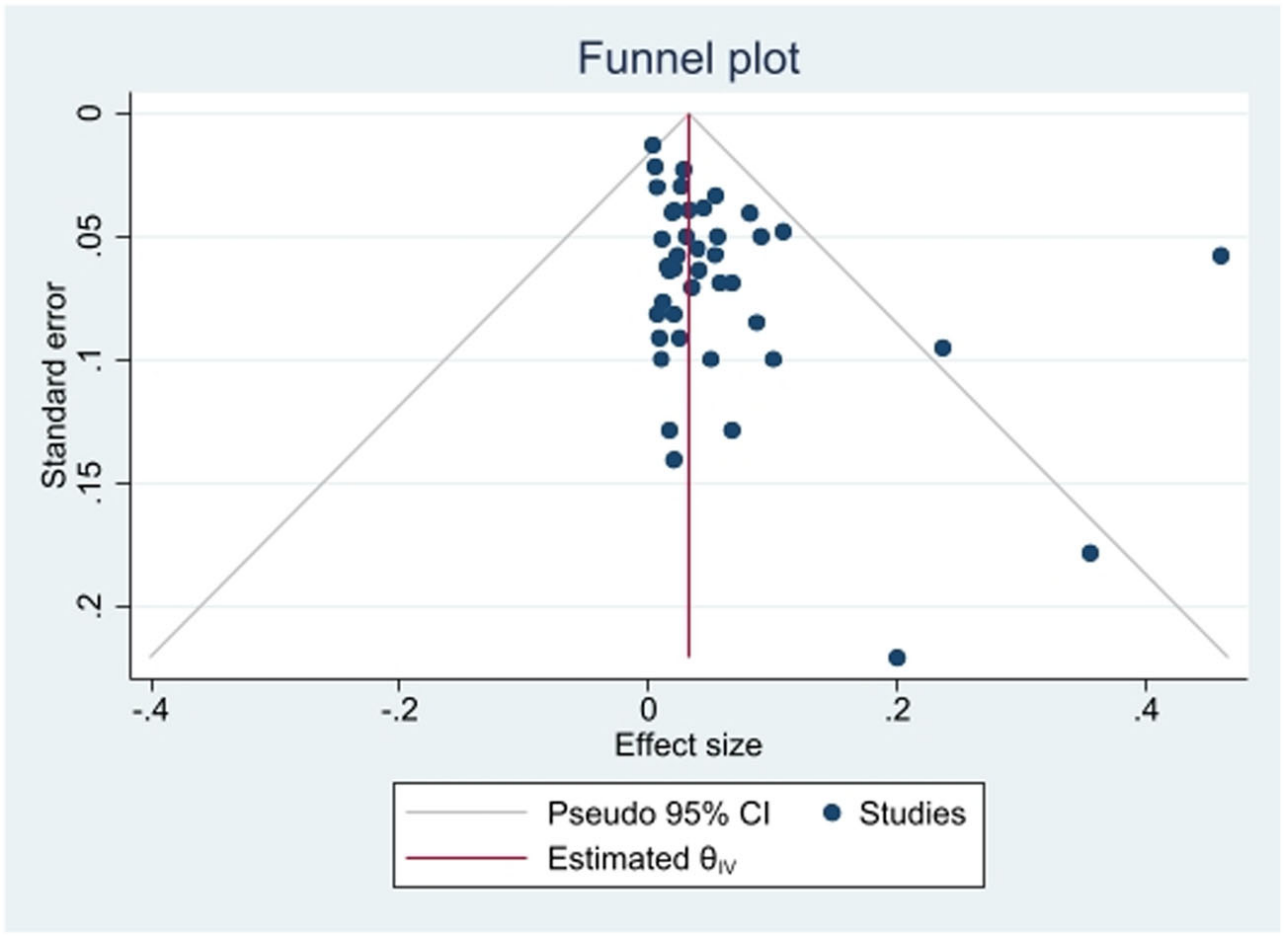
Funnel plot for identification of publication bias about the prevalence of *S*. Typhimurium in total isolates.

Funnel plots (Figure 3) showed publication bias for the prevalence result of *S*. Typhimurium in the total human, food, and animal isolates. Additionally, Begg's and Egger's tests were performed to quantitatively evaluate the publication biases. According to the results of Begg's test (*p* = 0.0640) and Egger's test (*p* = 0.0798), a significant publication bias was not observed. The sensitivity analysis indicated that excluding individual literature sources did not significantly alter the pooled effect estimate.

Our meta‐analysis revealed significant heterogeneity in the prevalence of *S*. Typhimurium among all isolates and *Salmonella* isolates. Various factors may contribute to this heterogeneity, including sample type, as differences in the types of samples analyzed (human, animal, and food) can influence prevalence due to varying exposure risks and transmission dynamics [79]. Geography also plays a role, with geographic differences in climate, agricultural practices, and public health policies potentially impacting the prevalence of *S*. Typhimurium. Additionally, population characteristics related to demographic factors can affect prevalence rates through differences in exposure and susceptibility [80, 81]. To further explore these influences, we conducted subgroup analyzes to assess how these variables affect the observed heterogeneity.

#### 3.2.2 Subgroup Meta‐Analysis of Prevalence of *S*. Typhimurium in Total Isolates

**Figure 4:**
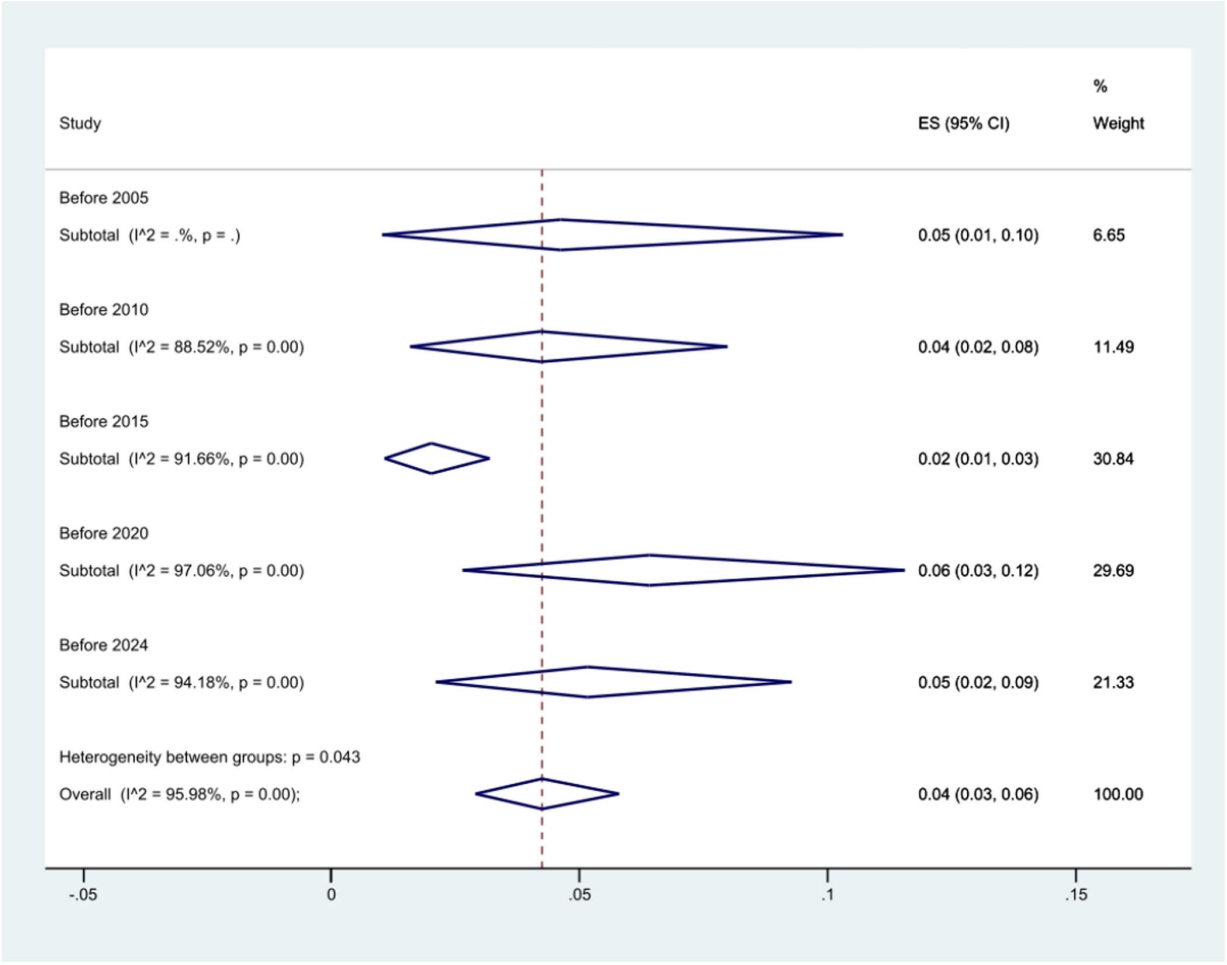
Forest plot showing the prevalence of *S*. Typhimurium in total isolates across different time intervals.

The analysis of *S*. Typhimurium prevalence over time indicated a nearly constant prevalence of this serovar across various time periods (*p* = 0.043) (Figure 4).

We categorized the origin of the isolates into samples isolated from humans, animals, meat, and food (including processed foods and eggs).

**Figure 5:**
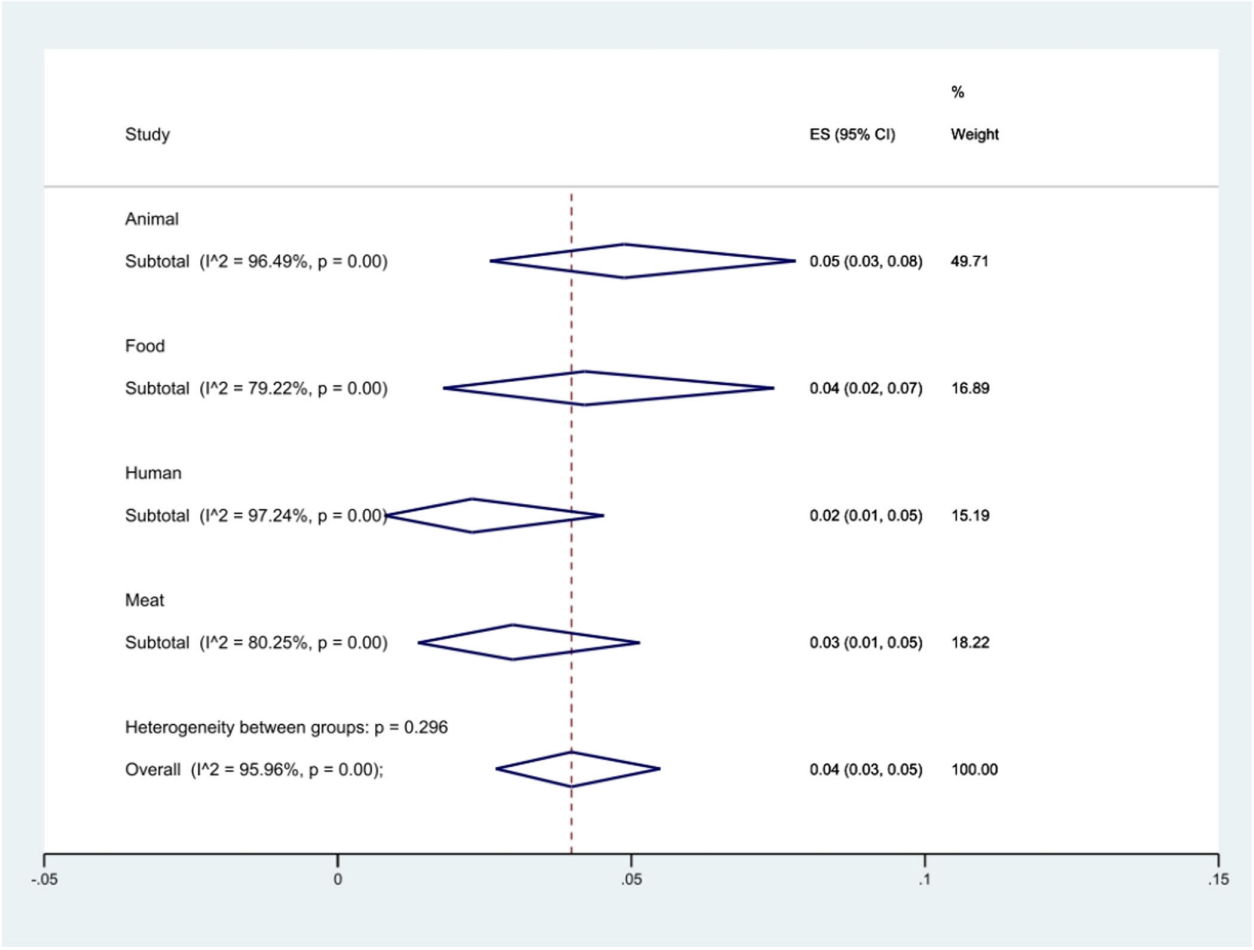
Forest plot showing the prevalence of *S*. Typhimurium in different sources.

Twenty‐one studies investigated the spread of *S*. Typhimurium in animal samples, eight studies focused on food, six studies on humans, and eight studies on meat samples. The highest prevalence was observed in *S*. Typhimurium isolates from animals at 5% (95% CI 3%–8%), and the lowest in isolates from humans at 2% (95% CI 1%–5%), (*p* = 0.296) (Figure 5).

**Figure 6:**
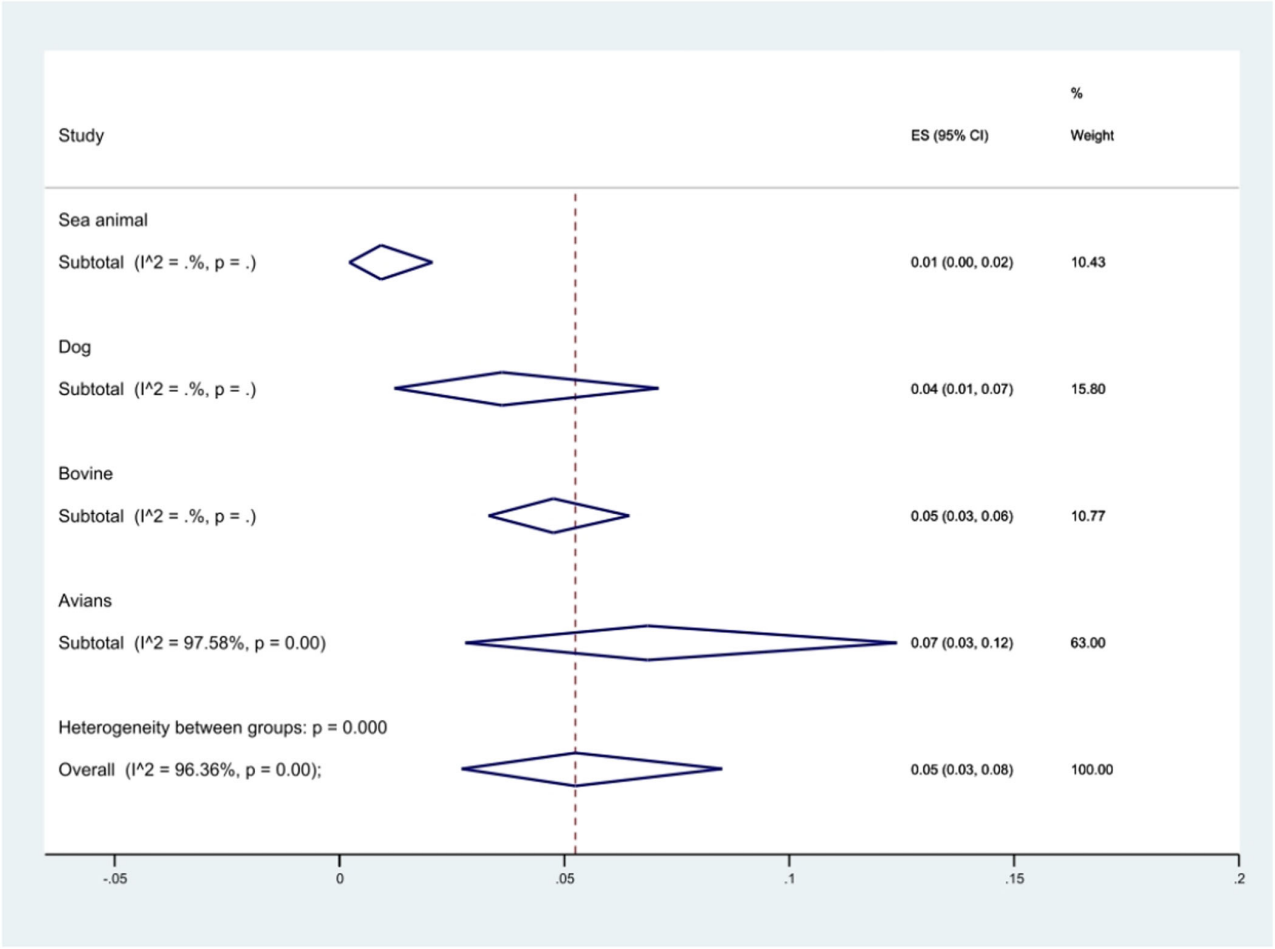
Forest plot showing the prevalence of *S*. Typhimurium in different animals.

Additionally, the prevalence of *S*. Typhimurium was analyzed based on the type of animal from which the samples were isolated. Twelve studies investigated the spread of *S*. Typhimurium in avian samples, two studies focused on bovine samples, three examined dogs, and two explored the presence of the bacteria in sea animals. The results showing that samples isolated from avians had the highest prevalence of *S*. Typhimurium at 7% (95% CI 3%–12%), (*p* < 0.001) (Figure 6).

**Figure 7:**
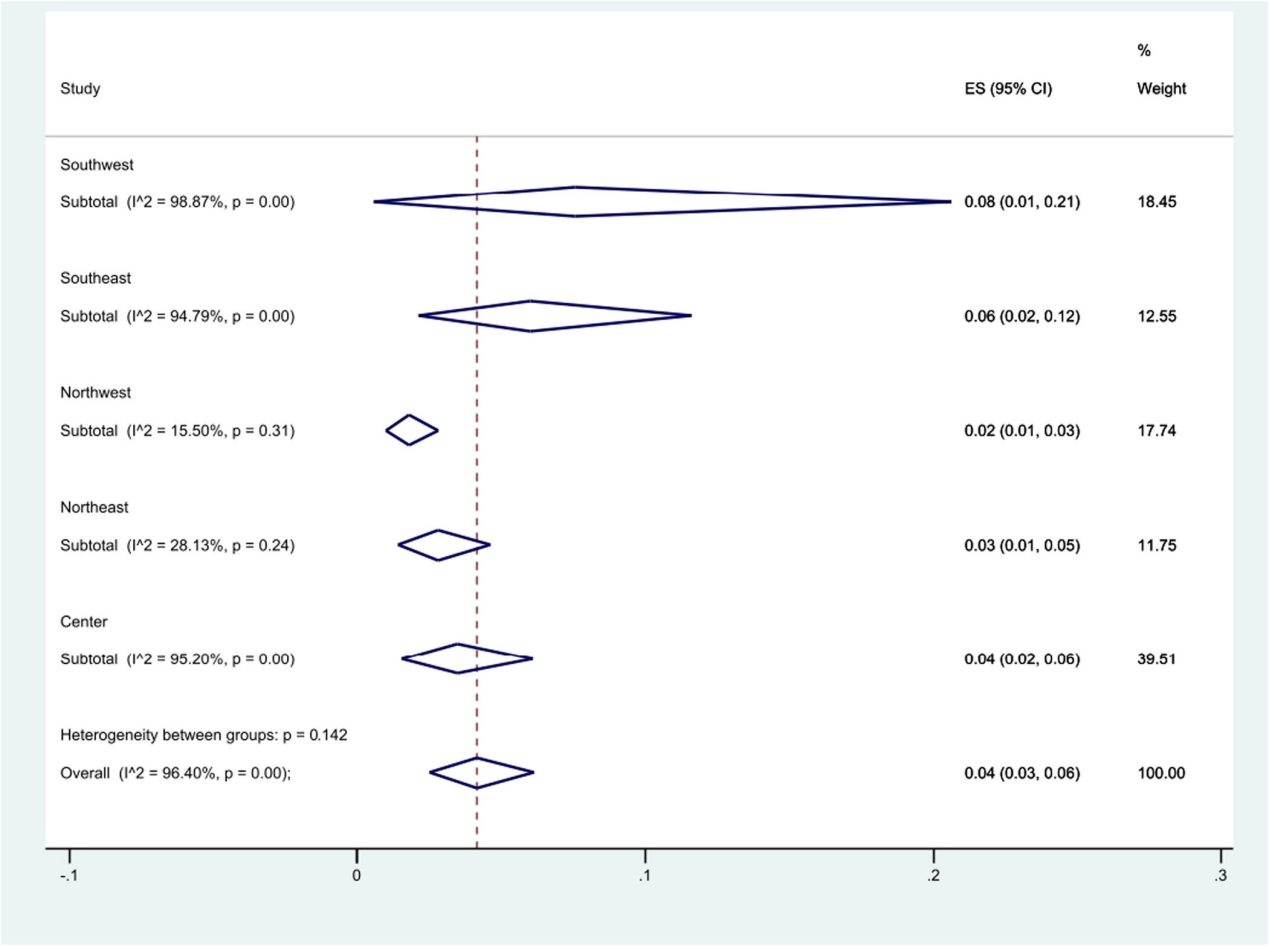
Forest plot showing the prevalence of *S*. Typhimurium in different regions of Iran.

Thirteen studies have examined the prevalence of *S*. Typhimurium in central cities of Iran. Additionally, four studies were conducted in the northeastern regions, six in the northwestern regions, four in the southeastern regions, and six in the southwestern regions. The southwestern region of Iran exhibited the highest prevalence of *S*. Typhimurium, recorded at 8% (95% CI 1%–21%), (*p* = 0.142) (Figure 7).

**Figure 8:**
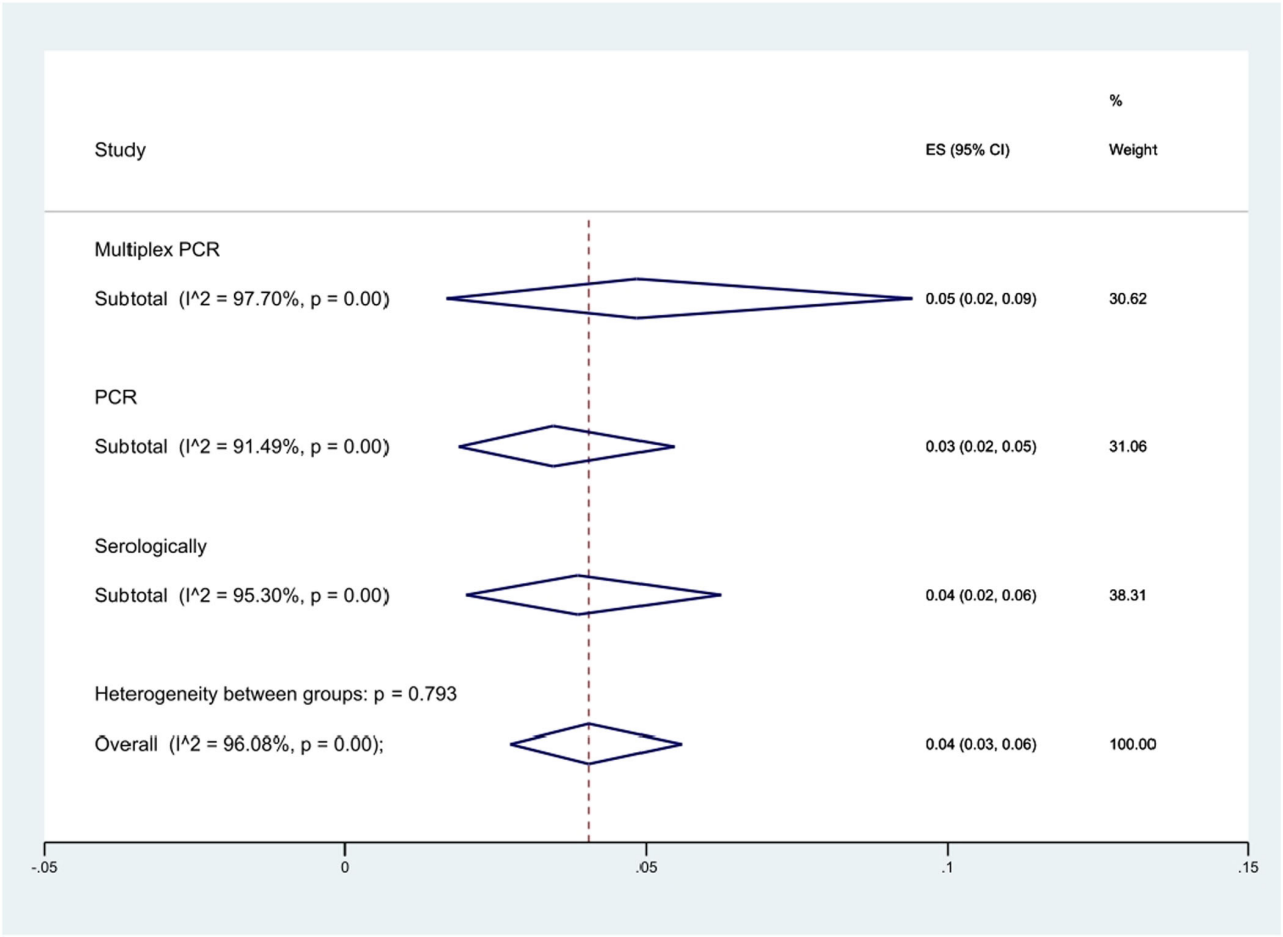
Forest plot showing the prevalence of *S*. Typhimurium in different detection methods.

We also examined the hypothesis that different detection methods could influence the prevalence of *S*. Typhimurium through subgroup analyzes. Thirteen studies used the multiplex PCR method, while another thirteen employed standard PCR, and sixteen studies utilized serological methods. The multiplex PCR method reported a prevalence of 5% (95% CI 2%–9%), higher than the other methods, although this difference was not statistically significant (*p* = 0.793) (Figure 8).

#### 3.2.3 Prevalence of *S*. Typhimurium in Isolated *Salmonella* Species

**Figure 9:**
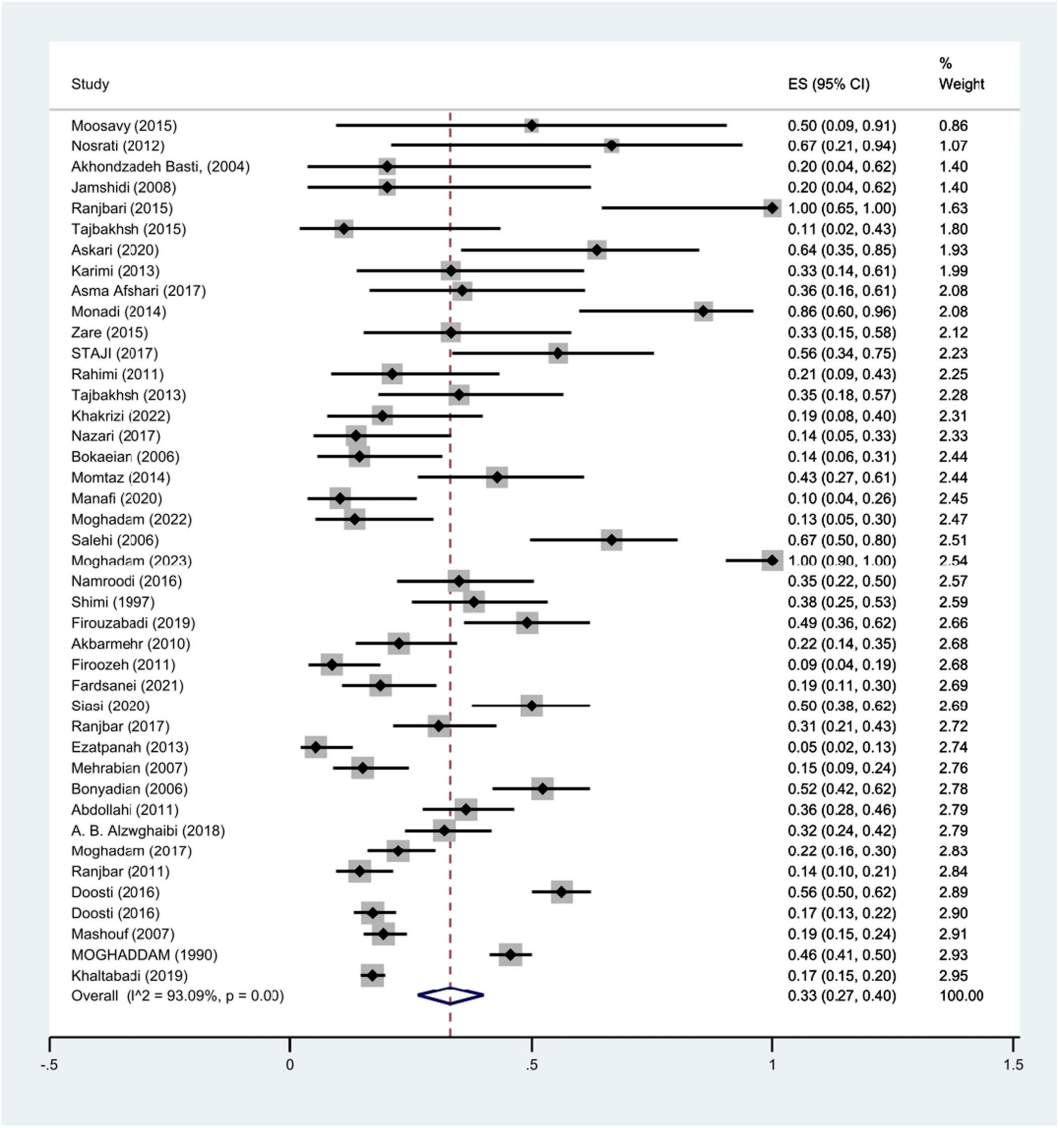
Forest plot showing the prevalence of *S*. Typhimurium in different *Salmonella* isolates.

**Figure 10:**
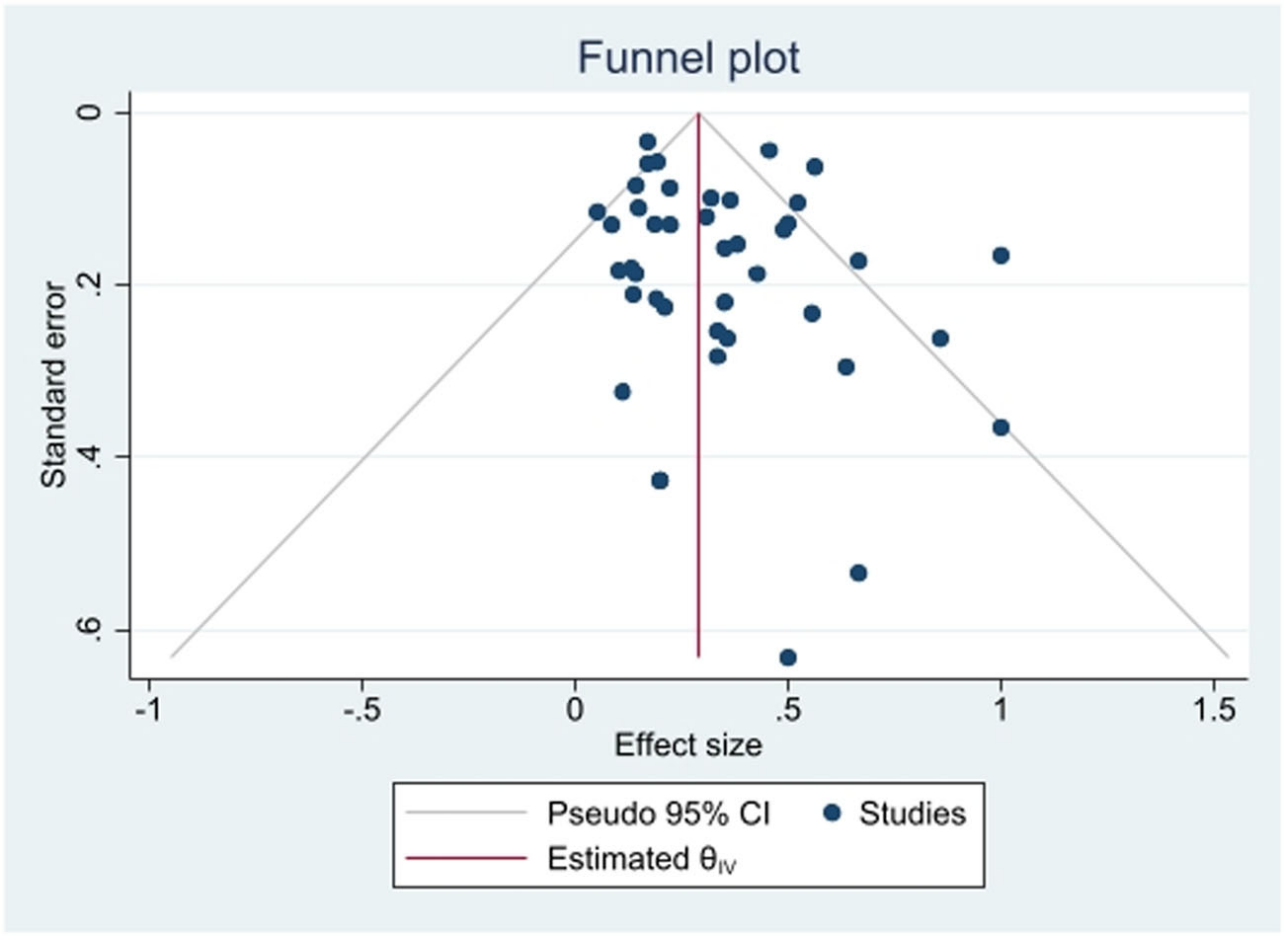
Funnel plot for identification of publication bias about the prevalence of *S*. Typhimurium in different *Salmonella* isolates.

Forty‐two studies provided information on the number of *S*. Typhimurium isolates among the *Salmonella* species that were isolated. The prevalence of *S*. Typhimurium was found to be 33% (95% CI: 27%–40%; I^2^ = 93.09%; *p* < 0.001) as illustrated in Figure 9. Funnel plots (Figure 10) displayed publication bias for the prevalence result of *S*. Typhimurium in the *Salmonella* species.

Furthermore, Begg's and Egger's tests were performed to quantitatively assess publication bias. The results indicated no significant publication bias, with Begg's test yielding a *p*‐value of 0.0994 and Egger's test a *p*‐value of 0.1768.

In our articles, funnel plots depict publication bias, while Begg's and Egger's tests show no significant bias. This difference may occur because small studies with large effect sizes can create visual asymmetries that may not be statistically significant [82]. The sensitivity analysis confirmed that the exclusion of individual literature sources did not materially affect the pooled effect estimate.

#### 3.2.4 Subgroup Meta‐Analysis of Prevalence of *S*. Typhimurium in Isolated *Salmonella* Species

**Figure 11:**
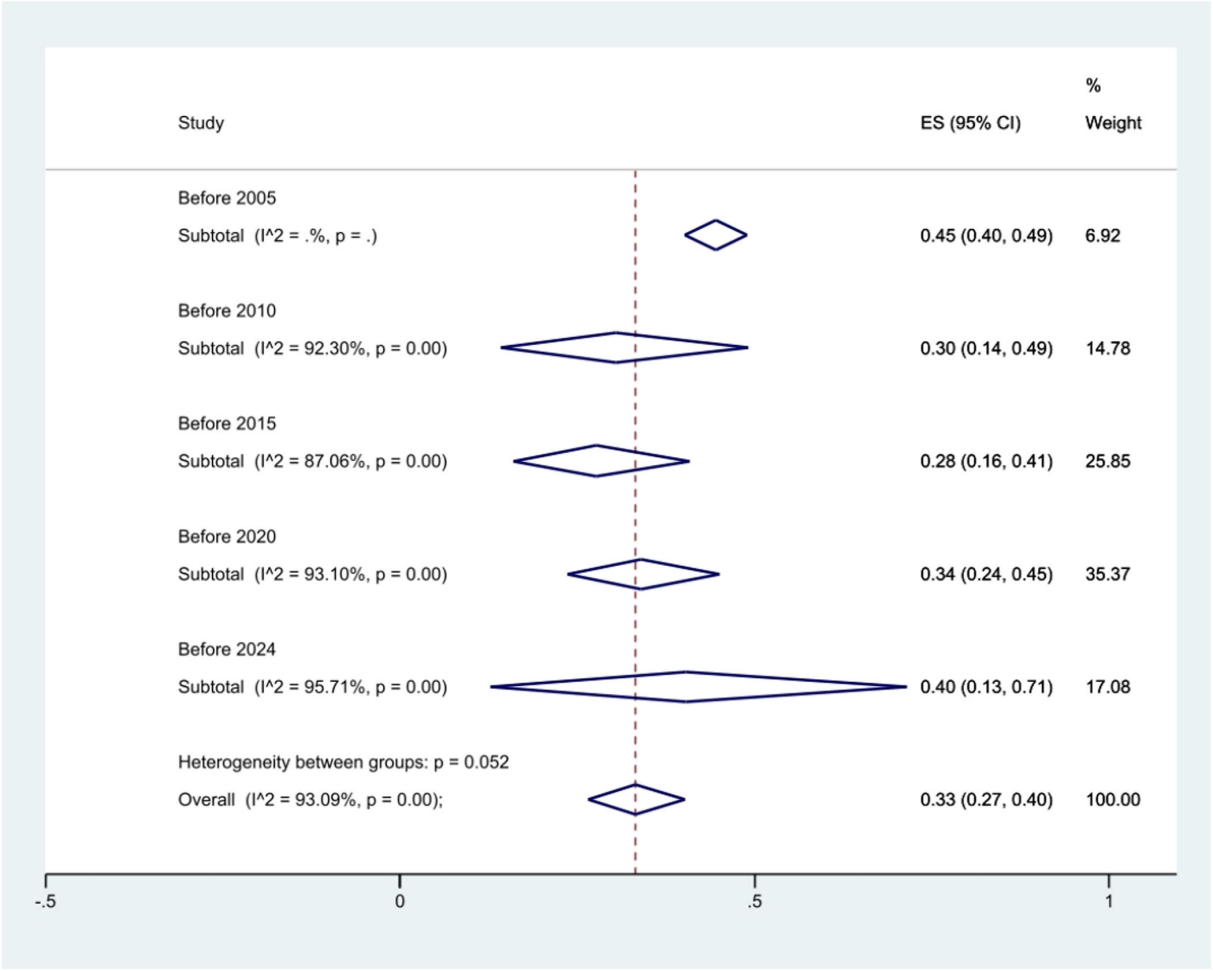
Forest plot showing the prevalence of *S*. Typhimurium in different *Salmonella* species isolates across different time intervals.

The analysis of *S*. Typhimurium prevalence over time indicated a nearly constant prevalence of this serovar across various time periods (*p* = 0.052) (Figure 11).

**Figure 12:**
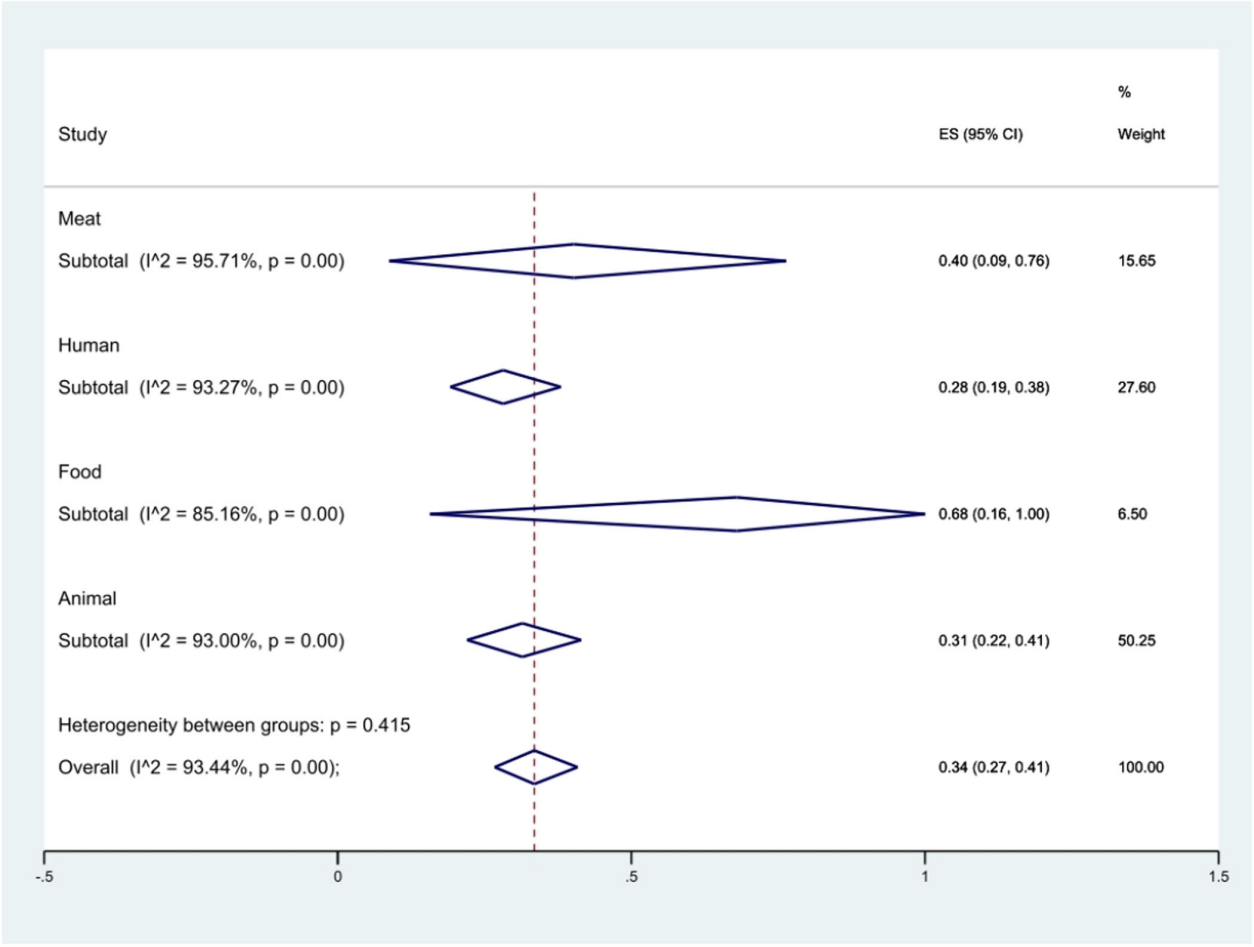
Forest plot showing the prevalence of *S*. Typhimurium in different *Salmonella* isolates in different sources.

Twenty‐one studies investigated the prevalence of *S*. Typhimurium in animal samples, four studies in food, ten studies in humans, and seven studies in meat samples. The highest prevalence was observed in *S*. Typhimurium isolated from food at 68% (95% CI 16%–100%), and the lowest in isolates isolated from humans at 28% (95% CI 19%–38%) (*p* = 0.415) as shown in Figure 12.

## 4 Discussion

To the best of our knowledge, there are limited systematic reviews investigating the prevalence of *S*. Typhimurium in Iran. Therefore, we systematically reviewed the published literature and conducted a meta‐analysis to determine the overall prevalence of *S*. Typhimurium in foods, animals, and humans in Iran.

Recent reports indicate a rising prevalence of *Salmonella* strains resistant to antimicrobial agents, which is a significant public health concern [83]. The application of antimicrobial agents in various environments creates conditions that favor the survival of antibiotic‐resistant pathogens [84]. The common practice of administering antimicrobial agents to domestic livestock for disease prevention, treatment, and growth promotion significantly contributes to the development of antibiotic‐resistant bacteria, which can then be transmitted to humans through the food chain [85, 86]. Most infections caused by antimicrobial‐resistant *S*. Typhimurium are linked to the consumption of contaminated animal‐derived foods [87].

Our study revealed that the overall prevalence of *S*. Typhimurium was 4% in all human and food samples. Furthermore, our analysis indicated that the level of infection with *S*. Typhimurium in animals was higher than in other samples, highlighting the importance of monitoring animals to prevent infections caused by this bacterium. The prevalence of *S*. Typhimurium among *Salmonella* species isolated from different sources was 33%, demonstrating the relatively high prevalence of this serovar among *Salmonella* species and emphasizing its importance.

A study conducted by Sinwat examined clinical samples from individuals working in the meat‐handling industry in Thailand and the Lao People's Democratic Republic. The findings revealed a concerning prevalence of *Salmonella*, with 34.6% of samples testing positive in Thailand and 47.4% in the Lao PDR. Further molecular analysis of these isolates identified *S*. Typhimurium as the predominant serotype, accounting for 34% of isolates in Thailand and 20.6% in the Lao PDR [88]. This highlights a potential risk for Invasive non‐typhoidal *Salmonella* (iNTS) disease in these regions [88]. In a 6‐year study in Colombia conducted by Rodríguez, 32.5% of *S. enterica* isolates sourced from blood and fecal samples were identified as *S*. Typhimurium [89]. Furthermore, literature indicates that *S. enterica* serovar Typhimurium is frequently linked to iNTS cases in sub‐Saharan Africa, underscoring the global significance of this serotype in public health concerns [90, 91].

Our analysis reveals that *S*. Typhimurium is the most prevalent serovar among *Salmonella* species in food samples. Alarmingly, 68% of the contaminated foods tested positive for this serovar. Additionally, 28% of human salmonellosis infections were caused by *S*. Typhimurium, underscoring the need for proper food screening and hygiene to prevent human infections. A meta‐analysis conducted by Ferrari et al. indicated that serovar Typhimurium is the most common worldwide [16]. Studies also showed that serovar Typhimurium ranks second in prevalence in Europe and third in the United States [92].

According to the predictions by the Organization for Economic Co‐operation and Development and the Food and Agricultural Organization (OECD‐FAO), factors such as low production costs, short production cycles, high feed conversion ratios, and low product prices are the main reasons for choosing poultry meat for producers and consumers [93]. A meta‐analysis by Ferrari et al. showed that serovars Typhimurium and Sofia are the most common serovars in poultry in North America and Oceania [16]. The meta‐analysis conducted by Sun et al. showed that serovar Typhimurium was the third most common serovar isolated from poultry in China, with a prevalence of 9.1% [94]. Paião et al. reported that in Brazil, *S*. Enteritidis and *S*. Typhimurium were found in 12% and 3% of broiler chicken samples, respectively [95]. In a study conducted in Turkey, Mutluer et al. found that 27.5% of broiler carcasses were contaminated with *Salmonella* spp. Among their isolates, serotyping results indicated that 25.5% were identified as *S*. Typhimurium [96]. Aury et al. investigated *Salmonella* in turkeys in France, identifying the five most prevalent serotypes: *S*. Derby (29.2%), *S*. Enteritidis (11.7%), *S*. Typhimurium (10.1%), *S*. Hadar (8.4%), and *S*. Mbandaka (6.8%) [97]. In a study by Kanaan, 150 raw and frozen poultry meat samples were collected from various retail markets in Iraq. Of these, 19 samples tested positive for *Salmonella*, with *S*. Enteritidis accounting for 63.2% and *S*. Typhimurium for 36.8% [98]. Our study also revealed that among the tested animals, avian had the highest prevalence of Typhimurium serovar at 7%, indicating the need for more effective intervention strategies during processing to control the quality and safety of poultry products in Iran.

In terms of geographical distribution, the prevalence of *S*. Typhimurium was highest in southwestern region. Farming practices and the presence of infected animals can significantly influence *Salmonella* prevalence [99]. Additionally, the elevated levels of *S*. Typhimurium in these areas may be attributed to inadequate sanitation conditions, including the use of contaminated equipment, poor water quality, high humidity in operational areas, personnel hygiene during manual processing, and airborne contamination during slaughter [100, 101]. Therefore, consistent monitoring of *S*. Typhimurium prevalence and food markets is essential for effective control of salmonellosis in these regions.

This study provides valuable insights into the prevalence of *S*. Typhimurium; however, it has limitations. These include the geographical dispersion of the included studies, a limited number of studies conducted in specific provinces, and a notable absence of research on the prevalence of this serotype in several provinces of Iran. To address these gaps, we conducted an investigation into the prevalence of *S*. Typhimurium, aiming to encompass a larger number of studies across five distinct regions of Iran.

In conclusion, our data confirm that *S*. Typhimurium has a high prevalence in animal and food isolates, and 28% of the *Salmonella* isolated from humans is Typhimurium. These results highlight the importance of further research to understand the prevalence of this microorganism in different regions, as well as increased monitoring in regions with high prevalence to prevent diseases caused by this bacterium.

## Author Contributions

**Negar Narimisa:** conceptualization, investigation, writing – original draft, writing – review and editing, software. **Shabnam Razavi:** methodology, validation, visualization, writing – review and editing. **Faramarz Masjedian Jazi:** data curation, supervision, resources, writing – review and editing.

## Ethics Statement

The authors have nothing to report.

## Consent

The authors have nothing to report.

## Conflicts of Interest

The authors declare no conflicts of interest.

## Transparency Statement

The lead author Faramarz Masjedian Jazi affirms that this article is an honest, accurate, and transparent account of the study being reported; that no important aspects of the study have been omitted; and that any discrepancies from the study as planned (and, if relevant, registered) have been explained.

## Data Availability

The data that supports the findings of this study are available in the supporting material of this article. All the data in this review are included in the article.

## References

[1] H. H. Abulreesh , Salmonellae in the Environment (InTech, 2012).

[2] N. Narimisa , S. Razavi , A. Khoshbayan , S. Gharaghani , and F. M. Jazi , “Targeting Lon Protease to Inhibit Persister Cell Formation in Salmonella Typhimurium: A Drug Repositioning Approach,” Frontiers in Cellular and Infection Microbiology 14 (2024): 1427312. 39301287 10.3389/fcimb.2024.1427312 PMC11410781

[3] S. Yan , W. Zhang , C. Li , et al., “Serotyping, MLST, and Core Genome MLST Analysis of Salmonella Enterica From Different Sources in China During 2004–2019,” Frontiers in Microbiology 12 (2021): 688614. 34603224 10.3389/fmicb.2021.688614 PMC8481815

[4] N. Narimisa , F. Amraei , B. S. Kalani , and F. M. Jazi , “Evaluation of Gene Expression and Protein Structural Modeling Involved in Persister Cell Formation in Salmonella Typhimurium,” Brazilian Journal of Microbiology 52 (2021): 207–217. 33125683 10.1007/s42770-020-00388-w PMC7966607

[5] C. A. Gómez‐Aldapa , M. R. Torres‐Vitela , A. Villarruel‐López , and J. Castro‐Rosas , “The Role of Foods in Salmonella Infections,” in Salmonella‐A Dangerous Foodborne Pathogen (2012), 21–46.

[6] G. L. Popa and M. I. Popa , “Salmonella spp. Infection‐A Continuous Threat Worldwide,” Germs 11, no. 1 (2021): 88–96. 33898345 10.18683/germs.2021.1244 PMC8057844

[7] R. K. Gast and R. E. Porter, Jr. , “Salmonella Infections,” in Diseases of Poultry (2020), 717–753.

[8] M. E. Pearce , G. C. Langridge , A. C. Lauer , K. Grant , M. C. J. Maiden , and M. A. Chattaway , “An Evaluation of the Species and Subspecies of the Genus Salmonella With Whole Genome Sequence Data: Proposal of Type Strains and Epithets for Novel S. Enterica Subspecies VII, VIII, IX, X, and XI,” Genomics 113, no. 5 (2021): 3152–3162. 34242711 10.1016/j.ygeno.2021.07.003 PMC8426187

[9] P. Branchu , M. Bawn , and R. A. Kingsley , “Genome Variation and Molecular Epidemiology of Salmonella Enterica Serovar Typhimurium Pathovariants,” Infection and Immunity 86, no. 8 (2018): e00079‐18, 10.1128/iai.00079-18. 29784861 PMC6056856

[10] V. Sangal . Multilocus Sequence Typing Analyses of Salmonella Enterica Subspecies Enterica. 2009.

[11] O. Gal‐Mor , E. C. Boyle , and G. A. Grassl , “Same Species, Different Diseases: How and Why Typhoidal and Non‐Typhoidal Salmonella Enterica Serovars Differ,” Frontiers in Microbiology 5 (2014): 391. 25136336 10.3389/fmicb.2014.00391 PMC4120697

[12] R. Balasubramanian , J. Im , J.‐S. Lee , et al., “The Global Burden and Epidemiology of Invasive Non‐Typhoidal Salmonella Infections,” Human Vaccines and Immunotherapeutics 15, no. 6 (2019): 1421–1426. 30081708 10.1080/21645515.2018.1504717 PMC6663144

[13] N. Narimisa , S. Razavi , and F. Masjedian Jazi , “Prevalence of Antibiotic Resistance in Salmonella Typhimurium Isolates Originating From Iran: A Systematic Review and Meta‐Analysis,” Frontiers in Veterinary Science 11 (2024): 1388790. 38860007 10.3389/fvets.2024.1388790 PMC11163077

[14] J. A. Wagenaar , R. S. Hendriksen , and J. J. Carrique‐Mas , “Practical Considerations of Surveillance of Salmonella Serovars Other Than Enteridis and Typhimurium: ‐En‐ ‐Fr‐ Considérations Pratiques Sur La Surveillance Des Sérovars De Salmonella Hors Enteritidis Et Typhimurium ‐Es‐ Consideraciones Prácticas En Torno Ala Vigilancia De Serovares De Salmonella Distintos De Enteritidis Y Typhimurium,” Revue Scientifique et Technique de l'OIE 32, no. 2 (2013): 509–519. 10.20506/rst.32.2.2244 24547654

[15] G. Arya , R. Holtslander , J. Robertson , et al., “Epidemiology, Pathogenesis, Genoserotyping, Antimicrobial Resistance, and Prevention and Control of Non‐Typhoidal Salmonella Serovars,” Current Clinical Microbiology Reports 4 (2017): 43–53.

[16] R. G. Ferrari , D. K. A. Rosario , A. Cunha‐Neto , S. B. Mano , E. E. S. Figueiredo , and C. A. Conte‐Junior , “Worldwide Epidemiology of Salmonella Serovars in Animal‐Based Foods: A Meta‐Analysis,” Applied and Environmental Microbiology 85, no. 14 (2019): e00591–19. 31053586 10.1128/AEM.00591-19 PMC6606869

[17] J. A. Naser , H. Hossain , M. S. R. Chowdhury , et al., “Exploring of Spectrum Beta Lactamase Producing Multidrug‐Resistant Salmonella Enterica Serovars in Goat Meat Markets of Bangladesh,” Veterinary and Animal Science 25 (2024): 100367. 38947184 10.1016/j.vas.2024.100367 PMC11214345

[18] M. M. Rahman , H. Hossain , M. S. R. Chowdhury , et al., “Molecular Characterization of Multidrug‐Resistant and Extended‐Spectrum β‐lactamases‐producing Salmonella Enterica Serovars Enteritidis and Typhimurium Isolated From Raw Meat In Retail Markets,” Antibiotics (USSR) 13, no. 7 (2024): 586. 10.3390/antibiotics13070586 PMC11274296 39061268

[19] G. L. Popa and M. I. Popa , “Salmonella Spp. Infection ‐ a Continuous Threat Worldwide,” Germs 11, no. 1 (2021): 88–96. 33898345 10.18683/germs.2021.1244 PMC8057844

[20] M. J. Page , J. E. McKenzie , P. M. Bossuyt , et al., “The Prisma 2020 Statement: An Updated Guideline for Reporting Systematic Reviews,” International Journal of Surgery 88 (2021): 105906. 33789826 10.1016/j.ijsu.2021.105906

[21] M. Ouzzani , H. Hammady , Z. Fedorowicz , and A. Elmagarmid , “Rayyan‐a Web and Mobile App for Systematic Reviews,” Systematic Reviews 5, no. 1 (2016): 210. 27919275 10.1186/s13643-016-0384-4 PMC5139140

[22] M.‐H. Moosavy , S. Esmaeili , F. Bagheri Amiri , E. Mostafavi , and T. Zahraei Salehi , “Detection of Salmonella Spp in Commercial Eggs in Iran,” Iranian Journal of Microbiology 7, no. 1 (2015): 50–54. 26644874 PMC4670468

[23] A. Nosrat , S. Azar , D. Mehrooz , T. Bahman , and F. Fatemeh , “Prevalence of Salmonella Enteritidis, Typhi and Typhimurium From Food Products in Mofid Hospital,” Journal of Research in Medical Sciences 36, no. 1 (2012): 43–48.

[24] A. A. Basti , T. Z. Salehi , and S. Bokaie , “Some Bacterial Pathogens in the Intestine of Cultivated Silver Carp and Common Carp,” Developments in Food Science 42: Elsevier (2004): 447–451.

[25] A. E. Jamshidi , M. Basami , and N. S. Afshari Identification of Salmonella spp. and Salmonella Typhimurium by a Multiplex PCR‐Based Assay From Poultry Carcasses in Mashhad‐Iran. 2009.

[26] R. Ali Ghorbani , N. Shahriar , Z. Abdollah , and R. Nazanin Ghorbani , “A Study of Salmonella Spp. Contamination in Egg of Ducks and Turkeys, Consumed in Fars Province,” Yafteh 17, no. 1 (2015): 104–109.

[27] T. Elaheh and M. Manouchehr , “Detection of Staphylococcus Areus and Salmonella Typimurium in Traditional and Industrial Olivie Salads in Shahrekord City,” Journal of Food Microbiology 2, no. 1 (2015): 39–48.

[28] S. M. R. Nader Askari and K. Amini , “Identification, Serotyping and Determination of Antibiotic Resistance of Salmonella Isolates Obtained From Stray Dogs in Tehran,” Journal of Veterinary Microbiology 16, no. 1 (2020): 43–52.

[29] H. Karimi Darehabi , F. Esmailneshad , and K. Ebrahimi Mohammadi , “Contamination of Fresh Beef to Salmonella Typhimurium and Salmonella Enteritidis in Sanandaj During 2012,” Quarterly Scientific–Research Journal Food Hygiene 2, no. 3 (2012): 41.

[30] A. Afshari , A. Baratpour , S. Khanzade , and A. Jamshidi , “Salmonella Enteritidis and Salmonella Typhimorium Identification in Poultry Carcasses,” Iranian Journal of Microbiology 10, no. 1 (2018): 45–50. 29922418 PMC6004630

[31] K. Monadim , M. Naghihaa , and M. Najafia , “Molecular Detection of Salmonella Serovar Isolated From Eggs,” Medical Laboratory Journal 9, no. 1 (2015): 17.

[32] P. Zare , H. Ghorbani‐Choboghlo , M. Tolouei , and J. Hadavi , “Evaluation of the Occurrence of Salmonella Serovars and Its Antibiotic Susceptibility in Apparently Healthy Domestic Animals in Rural Areas of East Azerbaijan Province,” Iranian Journal of Microbiology 10 (2016): 88–92.

[33] H. Staji , S. Rezaei , M. Rassouli , and S. Namroodi , “Prevalence and Genetic Characteristics of Salmonella Strains in Wild Mallard Ducks (*Anas platyrhynchos*) in Semnan Suburb, Iran,” Bulgarian Journal of Veterinary Medicine 20, no. 4 (2017): 348–356.

[34] E. Rahimi , A. Shakerian , and A. G. Falavarjani , “Prevalence and Antimicrobial Resistance of Salmonella Isolated From Fish, Shrimp, Lobster, and Crab in Iran,” Comparative Clinical Pathology 22 (2013): 59–62.

[35] F. Tajbakhsh , E. Tajbakhsh , E. Rahimi , and M. Momenii , “Determination of Antibiotic Resistance in Salmonella Spp Isolated From Raw Cow, Sheep and Goat's Milk in Chaharmahal va Bakhtiyari Provience, Iran,” Global Veterinaria 10 (2013): 681–685.

[36] A. Akbari Khakrizi , R. Yahyaraeyat , I. Ashrafi Tamai , B. Beikzadeh , and T. Zahraei Salehi , “Prevalence Assessment of Salmonella Serovars in Apparently Healthy Pet Dogs in Tehran,” Iran. Iranian Journal of Veterinary Science and Technology 14, no. 2 (2022): 11–18.

[37] M. Marziyeh Nazari , R. Ebrahim , and S. Amir , “Salmonella Contamination of Camel Meat in Various Stages of Destruction in Isfahan and Chaharmahal va Bakhtiari,” Journal of Food Microbiology 4, no. 2 (2017): 29–36.

[38] B. Mohammad , H. M. Amir , and G. Roqiah , “An Investigation on Contamination of Poultries by Salmonella Species in Zahedan (South‐East Iran) During 2004,” Research Journal of Microbiology 5, no. 11 (2010): 1185–1188.

[39] M. Hasan , G. Mohsen , and M. Manouchehr , “Detection of Virulence Factors in Salmonella Typhimurium and Salmonella Enteritidis Serotypes Isolated From Chicken Meat in Chaharmahal va Bakhtiari Province of Iran,” Journal of Food Microbiology 1, no. 1 (2014): 17–22.

[40] L. Manafi , J. Aliakbarlu , and H. Dastmalchi Saei , “Antibiotic Resistance and Biofilm Formation Ability of Salmonella Serotypes Isolated From Beef, Mutton, and Meat Contact Surfaces at Retail,” Journal of Food Science 85, no. 8 (2020): 2516–2522. 32671849 10.1111/1750-3841.15335

[41] S. Moghadam , S. Moradi Bidhendi , and P. Khaki , “Molecular Identification of Salmonella Strains Isolated From Livestock in Alborz Province and Their Serotyping,” Iranian Journal of Medical Microbiology 16, no. 4 (2022): 305–311.

[42] T. Zahraei Salehi , H. Tadjbakhsh , N. Atashparvar , M. G. Nadalian , and M. R. Mahzounieh , “Detection and Identification of Salmonella Typhimurium in Bovine Diarrhoeic Fecal Samples by Immunomagnetic Separation and Multiplex PCR Assay,” Zoonoses and Public Health 54, no. 6–7 (2007): 231–236. 17803511 10.1111/j.1863-2378.2007.01061.x

[43] M. Nazari Moghadam , E. Rahimi , A. Shakerian , and H. Momtaz , “Prevalence of Salmonella Typhimurium and Salmonella Enteritidis Isolated From Poultry Meat: Virulence and Antimicrobial‐Resistant Genes,” BMC Microbiology 23, no. 1 (2023): 168. 37322421 10.1186/s12866-023-02908-8 PMC10268442

[44] S. Namroodi , H. Estaji , and M. Dehmordeh , “Frequency and Antimicrobial Resistance Pattern of Salmonella Spp in Asymptomatic Rural Dogs,” Journal of Mazandaran University of Medical Sciences 1, no. 1 (2018): 44–50.

[45] A. Shimi and A. Barin , “Salmonella in Cats,” Journal of Comparative Pathology 87, no. 2 (1977): 315–318. 858815 10.1016/0021-9975(77)90020-2

[46] A. Firouzabadi , D. Saadati , M. Najimi , and M. Jajarmi , “Prevalence and Related Factors of Salmonella spp. and Salmonella Typhimurium Contamination Among Broiler Farms in Kerman Province, Iran,” Preventive Veterinary Medicine 175 (2020): 104838. 31812008 10.1016/j.prevetmed.2019.104838

[47] J. Akbarmehr , T. Z. Salehi , and G. Brujeni , “Identification of Salmonella Isolated From Poultry by MPCR Technique and Evaluation of Their Hsp groEL Gene Diversity Based on the PCR‐RFLP Analysis,” African Journal of Microbiology Research 4, no. 15 (2010): 1594–1598.

[48] F. Firoozeh , F. Shahcheraghi , T. Zahraei Salehi , V. Karimi , and M. M. Aslani , “Antimicrobial Resistance Profile and Presence of Class I Integrongs Among Salmonella Enterica Serovars Isolated From Human Clinical Specimens in Tehran, Iran,” Iranian Journal of Microbiology 3, no. 3 (2011): 112–117. 22347592 PMC3279819

[49] F. Fardsanei , M. M. Soltan Dallal , T. Zahraei Salehi , M. Douraghi , M. Memariani , and H. Memariani , “Antimicrobial Resistance Patterns, Virulence Gene Profiles, and Genetic Diversity of Salmonella Enterica Serotype Enteritidis Isolated From Patients With Gastroenteritis in Various Iranian Cities,” Iranian Journal of Basic Medical Sciences 24, no. 7 (2021): 914–921. 34712421 10.22038/ijbms.2021.54019.12142 PMC8528249

[50] S. Elham , A. Bahareh , and M. Sedigheh , “Comparison of Virulence Genes (int, inv, spv (vir)) in Salmonella Typhimurium and Infantis From Clinical Cases by Multiplex PCR,” Iranian Journal of Infectious Diseases 25, no. 90 (2021): 27–36.

[51] R. Ranjbar , P. Elhaghi , and L. Shokoohizadeh , “Multilocus Sequence Typing of the Clinical Isolates of Salmonella Enterica Serovar Typhimurium in Tehran Hospitals,” Iranian Journal of Medical Sciences 42, no. 5 (2017): 443–448. 29234176 PMC5722961

[52] E. Ezatpanah , S. M. Bidhendi , P. Khaki , R. Ghaderi , E. S. Jasbi , and S. M. Far , “Isolation, Serotyping and Antibiotic‐Resistance Pattern of Isolated Salmonella From Chicken of Arak,” Iranian Veterinary Journal 9, no. 39 (2013): 88–96.

[53] S. Mehrabian and E. Jaberi , “Isolasion, Identification and Antimicrobial Resistance Patterns of Salmonella From Meat Products in Tehran,” Pakistan Journal of Biological Sciences 10, no. 1 (2006): 122–126. 10.3923/pjbs.2007.122.126 19069997

[54] M. Bonyadian , A. S. Ale , and F. A. Motahari Isolation and identification of Salmonellae From Chicken Carcasses in Processing Plants in Yazd Province, Central Iran. 2007.

[55] A. Abbas , N. Sohrab , K. Seyyed Amin , M. Mohammad Hassan , and A. Majid Naghdi Other , “Salmonella Enterica: Serotyping, Drug Resistance and Extended Spectrum of β‐Lactamase (ESBLs),” Journal of Advanced Biomedical Sciences 1, no. 1 (2011): 38.

[56] A. B. Alzwghaibi , R. Yahyaraeyat , B. N. Fasaei , A. G. Langeroudi , and T. Z. Salehi , “Rapid Molecular Identification and Differentiation of Common Salmonella Serovars Isolated From Poultry, Domestic Animals and Foodstuff Using Multiplex PCR Assay,” Archives of Microbiology 200 (2018): 1009–1016. 29627903 10.1007/s00203-018-1501-7

[57] M. Abolfazl and D. Shahram Nazarian , “Evaluation of Class 1, 2 and 3 Integrons in Clinical Salmonella Enteritidis Strains by Pcr Method. Jorjani Biomedicine,” Journal 5, no. 2 (2018): 1–10.

[58] R. Ranjbar , G. M. Giammanco , S. Farshad , P. Owlia , A. Aleo , and C. Mammina , “Serotypes, Antibiotic Resistance, and Class 1 Integrons in Salmonella Isolates From Pediatric Cases of Enteritis in Tehran, Iran,” Foodborne Pathogens and Disease 8, no. 4 (2011): 547–553. 21204690 10.1089/fpd.2010.0736

[59] A. Doosti , E. Mahmoudi , M.‐S. Jami , and A. Mokhtari‐Farsani , “Prevalence of aadA1, aadA2, aadb, strA and strB Genes and Their Associations With Multidrug Resistance Phenotype in Salmonella Typhimurium Isolated From Poultry Carcasses,” Thai Journal of Veterinary Medicine 46, no. 4 (2016): 691–697.

[60] A. Doosti , A. Zohoor , M. Chehelgerdi , and A. Mokhtari‐Farsani , “Distribution of TEM‐1 Gene in Salmonella Enterica Isolated From Poultry Carcasses in Iran,” Thai Journal of Veterinary Medicine 46, no. 1 (2016): 9–15.

[61] R. Yousefi‐Mashouf and A. Moshtaghi , “Frequency of Typhoidal and Non‐Typhoidal Salmonella Species and Detection of Their Drugs Resistance Patterns,” Journal of Research in Health Sciences 7, no. 1 (2023): 49–56. 23343871

[62] A. A. Farhoudi‐Moghaddam , M. Katouli , A. Jafari , M. A. Bahavar , M. Parsi , and F. Malekzadeh , “Antimicrobial Drug Resistance and Resistance Factor Transfer Among Clinical Isolates of Salmonellae in Iran,” Scandinavian Journal of Infectious Diseases 22, no. 2 (1990): 197–203. 2356442 10.3109/00365549009037902

[63] R. F. Khaltabadi , N. Shahrokhi , M. Ebrahimi‐Rad , and P. Ehsani , “Salmonella Typhimurium in Iran: Contribution of Molecular and IS 200 PCR Methods in Variants Detection,” PLoS One 14, no. 3 (2019): e0213726. 30865712 10.1371/journal.pone.0213726 PMC6415898

[64] A. Azizpour , “Prevalence and Antibiotic Resistance of Salmonella Serotypes in Chicken Meat of Ardabil, Northwestern Iran,” Iranian Journal of Medical Microbiology 15, no. 2 (2021): 232–246.

[65] M. Dilmaghani , M. Ahmadi , T. Zahraei Salehi , and A. Talebi , “The Analysis of groEL Gene in Salmonella Enterica Subspecies Enterica Serovar Typhimurium Isolated From Avians by PCR‐Restriction Fragment Length Polymorphism Method,” Veterinary Research Communications 35 (2011): 133–143. 21312060 10.1007/s11259-011-9460-3

[66] H. A. Halimi , H. A. Seifi , and M. Rad , “Bovine Salmonellosis in Northeast of Iran: Frequency, Genetic Fingerprinting and Antimicrobial Resistance Patterns of Salmonella Spp,” Asian Pacific Journal of Tropical Biomedicine 4, no. 1 (2014): 1–7. 24144122 10.1016/S2221-1691(14)60199-4 PMC3819487

[67] A. Jamshidi , G. A. Kalidari , and M. Hedayati , “Isolation and Identification of Salmonella Enteritidis and Salmonella Typhimurium From the Eggs of Retail Stores in Mashhad, Iran Using Conventional Culture Method and Multiplex PCR Assay,” Journal of Food Safety 30, no. 3 (2010): 558–568.

[68] M. A. Keshmiri , A. Nemati , M. Askari Badouei , I. Ashrafi Tamai , and T. Zahraei Salehi , “Clonal Relatedness and Antimicrobial Susceptibility of Salmonella Serovars Isolated From Humans and Domestic Animals in Iran: A One Health Perspective,” Iranian Journal of Veterinary Research 23, no. 2 (2022): 104–110. 36118610 10.22099/IJVR.2022.40594.5881 PMC9441156

[69] A. Nazer and G. Safari , “Bacterial Flora From Dead‐in‐Shell Chicken Embryos and Their Drug Resistance in Fars Province of Iran,” Indian Journal of Animal Sciences 64, no. 10 (1994): 1006–1009.

[70] Z. Rahimi , P. Ghajarbeygi , R. Mahmoudi , S. Mosavi , and A. Mehrabi , “The Prevalence of Salmonella Enteritidis in Packaged and Tray Eggs Samples, Qazvin, Iran,” Journal of Chemical Health Risks 13, no. 3 (2023): 1–13.

[71] R. Ranjbar and A. Mirzaee , “Determining of the Variety of Genotypes in Salmonella Typhimurium by ERIC‐PCR,” Journal of Babol University of Medical Sciences 15, no. 1 (2012): 51.

[72] E. Estabergi , K. Amini , B. Shojaee Saadi , and S. Eghdamian , “Molecular Study of Salmonella Typhimurium Integrons Gene Isolated From Food Sources and the Effect of Probiotic Bifidobacterium Bifidum on its Expression by Real Time PCR. Razi,” Journal of Medical Sciences 27, no. 7 (2020): 65–77.

[73] A. Moghadam and S. Nazarian Genotyping of Clinical Isolates of Salmonella Enterica Serovar Typhimurium From Medical Centers of Kerman Province Using ERICPCR Method. 2018.

[74] J. A. C. Sterne , A. J. Sutton , J. P. A. Ioannidis , et al., “Recommendations for Examining and Interpreting Funnel Plot Asymmetry in Meta‐Analyses of Randomised Controlled Trials,” BMJ 343 (2011): d4002. 21784880 10.1136/bmj.d4002

[75] C. B. Begg and M. Mazumdar , “Operating Characteristics of a Rank Correlation Test for Publication Bias,” Biometrics 50, no. 4 (1994): 1088–1101. 7786990

[76] L. M. Becker . The Effects of Exercise Versus Methylphenidate on Attention and Behavior in Children With Attention Deficit Hyperactivity Disorder, Predominantly Inattentive Type: The University of Alabama at Birmingham; 1997.

[77] S. Freeman and A. Sutton , Identifying Publication Bias in Meta‐analyses of Continuous Outcomes (Cochrane Training, 2020).

[78] N. A. Patsopoulos , E. Evangelou , and J. P. Ioannidis , “Sensitivity of Between‐Study Heterogeneity in Meta‐Analysis: Proposed Metrics and Empirical Evaluation,” International Journal of Epidemiology 37, no. 5 (2008): 1148–1157. 18424475 10.1093/ije/dyn065 PMC6281381

[79] D. Belina , Y. Hailu , T. Gobena , T. Hald , and P. M. K. Njage , “Prevalence and Epidemiological Distribution of Selected Foodborne Pathogens in Human and Different Environmental Samples in Ethiopia: A Systematic Review and Meta‐Analysis,” One Health Outlook 3, no. 1 (2021): 19. 34474688 10.1186/s42522-021-00048-5 PMC8414678

[80] E. Feczko and D. A. Fair , “Methods and Challenges for Assessing Heterogeneity,” Biological Psychiatry 88, no. 1 (2020): 9–17. 32386742 10.1016/j.biopsych.2020.02.015 PMC8404882

[81] T. Ruppar , “Meta‐Analysis: How to Quantify and Explain Heterogeneity?,” European Journal of Cardiovascular Nursing 19, no. 7 (2020): 646–652. 32757621 10.1177/1474515120944014

[82] J. A. C. Sterne and R. M. Harbord , “Funnel Plots in Meta‐Analysis,” Stata Journal: Promoting Communications on Statistics and Stata 4 (2004): 127–141.

[83] F. Medalla , W. Gu , C. R. Friedman , et al., “Increased Incidence of Antimicrobial‐Resistant Nontyphoidal Salmonella Infections, United States, 2004–2016,” Emerging Infectious Diseases 27, no. 6 (2021): 1662–1672. 34013877 10.3201/eid2706.204486 PMC8153855

[84] L. Serwecińska , “Antimicrobials and Antibiotic‐Resistant Bacteria: A Risk to the Environment and to Public Health,” Water 12, no. 12 (2020): 3313.

[85] F. M. Aarestrup . “The Livestock Reservoir for Antimicrobial Resistance: A Personal View on Changing Patterns of Risks, Effects of Interventions and the Way Forward,” Philosophical Transactions of the Royal Society, B: Biological Sciences 370, no. 1670 (2015): 20140085. 10.1098/rstb.2014.0085 PMC4424434 25918442

[86] J. Bengtsson‐Palme , E. Kristiansson , and D. G. J. Larsson , “Environmental Factors Influencing the Development and Spread of Antibiotic Resistance,” FEMS Microbiology Reviews 42, no. 1 (2018): fux053. 29069382 10.1093/femsre/fux053 PMC5812547

[87] T. Hald , D. M. A. Lo Fo Wong , and F. M. Aarestrup , “The Attribution of Human Infections With Antimicrobial Resistant Salmonella Bacteria in Denmark to Sources of Animal Origin,” Foodborne Pathogens and Disease 4, no. 3 (2007): 313–326. 17883315 10.1089/fpd.2007.0002

[88] N. Sinwat , S. Angkittitrakul , K. F. Coulson , F. M. I. R. Pilapil , D. Meunsene , and R. Chuanchuen , “High Prevalence and Molecular Characteristics of Multidrug‐Resistant Salmonella in Pigs, Pork and Humans in Thailand and Laos Provinces,” Journal of Medical Microbiology 65, no. 10 (2016): 1182–1193. 27542886 10.1099/jmm.0.000339

[89] E. C. Rodríguez , P. Díaz‐Guevara , J. Moreno , et al., “Vigilancia Por Laboratorio De Salmonella Enterica En Casos Clínicos Humanos En Colombia 2005 a 2011,” Enfermedades Infecciosas y Microbiología Clínica 35, no. 7 (2017): 417–425. 27038678 10.1016/j.eimc.2016.02.023

[90] E. A. Reddy , A. V. Shaw , and J. A. Crump , “Community‐Acquired Bloodstream Infections in Africa: A Systematic Review and Meta‐Analysis,” Lancet Infectious Diseases 10, no. 6 (2010): 417–432. 20510282 10.1016/S1473-3099(10)70072-4 PMC3168734

[91] S. M. Graham , “Nontyphoidal Salmonellosis in Africa,” Current Opinion in Infectious Diseases 23, no. 5 (2010): 409–414. 20736739 10.1097/QCO.0b013e32833dd25d

[92] The European Union Summary Report on Antimicrobial Resistance in Zoonotic and Indicator Bacteria From Humans, Animals and Food in 2015,” EFSA Journal 15, no. 2 (2017): e04694. 32625402 10.2903/j.efsa.2017.4694 PMC7009883

[93] OECD/FAO . OECD‐FAO Agricultural Outlook 2020–2029. OECD. 2020.

[94] T. Sun , Y. Liu , X. Qin , et al., “The Prevalence and Epidemiology of Salmonella in Retail Raw Poultry Meat in China: A Systematic Review and Meta‐Analysis,” Foods 10, no. 11 (2021): 2757. 34829037 10.3390/foods10112757 PMC8622452

[95] F. G. Paião , L. G. A. Arisitides , L. S. Murate , G. T. Vilas‐Bôas , L. A. Vilas‐Boas , and M. Shimokomaki , “Detection of Salmonella spp, Salmonella Enteritidis and Typhimurium in Naturally Infected Broiler Chickens by a Multiplex PCR‐Based Assay,” Brazilian Journal of Microbiology 44 (2013): 37–42. 24159281 10.1590/S1517-83822013005000002 PMC3804175

[96] B. Mutluer , B. Yargülü , M. Hartung , and I. Erol Incidence and Serovar Distribution of Salmonella in Market Broilers in Turkey. 1992.

[97] K. Aury , M. Chemaly , I. Petetin , et al., “Prevalence and Risk Factors for Salmonella Enterica Subsp. Enterica Contamination in French Breeding and Fattening Turkey Flocks at the End of the Rearing Period,” Preventive Veterinary Medicine 94, no. 1–2 (2010): 84–93. 20044159 10.1016/j.prevetmed.2009.12.002

[98] M. H. G. Kanaan , “Prevalence and Antimicrobial Resistance of Salmonella Enterica Serovars Enteritidis and Typhimurium Isolated From Retail Chicken Meat in Wasit Markets, Iraq,” Veterinary World 16, no. 3 (2023): 455–463. 37041841 10.14202/vetworld.2023.455-463 PMC10082727

[99] A. E. Telli , Y. Biçer , N. Telli , C. Güngör , G. Türkal , and O. N. Ertaş , “Pathogenic *Escherichia coli* and Salmonella spp. in Chicken Carcass Rinses: Isolation and Genotyping by ERIC‐PCR,” Pakistan Veterinary Journal 42, no. 4 (2022): 493–498.

[100] A. Shah and N. Korejo , “Antimicrobial Resistance Profile of Salmonella Serovars Isolated From Chicken Meat,” Journal of Veterinary and Animal Sciences 2 (2012): 40–46.

[101] S. Balakrishnan , A. Sangeetha , and M. Dhanalakshmi , “Prevalence of Salmonella in Chicken Meat and its Slaughtering Place From Local Markets in Orathanadu, Thanjavur District, Tamil Nadu,” Journal of Entomology and Zoology Studies 6, no. 2 (2018): 2468–2471.

